# A Case of Malaria-Associated Hemophagocytic Lymphohistiocytosis

**DOI:** 10.7759/cureus.28386

**Published:** 2022-08-25

**Authors:** Mohamed Ramzi Almajed, Renato Cerna-Viacava, Jennifer Priessnitz, Naoshin Khan, Marcus Zervos

**Affiliations:** 1 Internal Medicine, Henry Ford Hospital, Detroit, USA; 2 School of Medicine, Wayne State University, Detroit, USA; 3 Infectious Diseases, Henry Ford Hospital, Detroit, USA

**Keywords:** international and travel medicine, hlh, hemophagocytic lymphohistiocytosis, plasmodium falciparum, malaria

## Abstract

Hemophagocytic lymphohistiocytosis (HLH) is an inflammatory syndrome of inappropriate and excessive immune system activation. It often occurs in the setting of viral, bacterial, fungal, and parasitic infections. HLH associated with malaria is very rare, and literature on this association is limited. Significant overlap exists between these two conditions, which makes the diagnosis of HLH superimposed on malaria difficult. We present a case of a patient who recently traveled from Djibouti and was diagnosed with Plasmodium falciparum malaria. She had a transient improvement in response to antimalarial therapy followed by clinical deterioration. This prompted further investigations that revealed the diagnosis of HLH, which was confirmed by an elevated soluble interleukin-2 receptor CD25 (sCD25) level, a specific marker of HLH. Most patients recover with antimalarial therapy, supportive care, and monitoring, whereas some patients require immunosuppressive therapy. Maintaining a high index of suspicion for HLH-associated malaria in at-risk patients allows for early identification and management.

## Introduction

Hemophagocytic lymphohistiocytosis (HLH) is a syndrome of inappropriate and excessive immune system activation. Dysfunction of natural killer cells and cytotoxic lymphocytes allows for macrophage hyperactivation, causing excess cytokine release, tissue damage, and multiorgan failure [1]. Conditions that trigger immune activation are often implicated in the development of HLH; these include infectious, autoimmune, metabolic, and neoplastic etiologies [2-3].

Malarial infections by Plasmodium parasites have been identified as a rare trigger of HLH; however, literature on this association is limited. The pathogenesis is unclear but likely involves an interplay between a patient's genetic susceptibility and a persistent and inappropriate immune response induced by the parasite. Diagnosing HLH in the setting of malaria is challenging as significant overlap exists between the clinical features and laboratory findings of these two entities. We present a case of HLH associated with Plasmodium falciparum malaria.

## Case presentation

A 32-year-old Yemeni woman presented to the hospital with complaints of persistent fever and generalized weakness for 10 days. She had myalgia, arthralgia, nausea, vomiting, and non-bloody diarrhea. She was mildly hypotensive with jaundice and marked splenomegaly. Her eldest son, an 11-year-old boy, was experiencing similar symptoms and had been admitted to another healthcare facility.

The patient was born in Yemen and moved to Djibouti later in life. She spent five years in Djibouti, during which she applied for immigration to the United States of America. She had arrived in Michigan, USA, from Djibouti three weeks prior to her presentation. Yemen is a country in the Middle East that is located in the south of the Arabian Peninsula; its population has a low socioeconomic status, and access to healthcare is limited. Djibouti is a country in East Africa that shares borders with Eretria, Ethiopia, and Somalia. Malaria is endemic in both Yemen and Djibouti, and efforts to combat malaria in these countries have been unsuccessful; they continue to be regarded as areas with a high local malarial transmission [4-6].

In the hospital, the patient's laboratory workup revealed a normal white cell count with anemia, thrombocytopenia, and elevated ferritin. Given the clinical picture and epidemiological risk factors, a malaria smear was ordered, which showed malaria parasites present with frequent ring forms and gametocytes consistent with Plasmodium falciparum species; parasitemia was estimated at 3%. Further infectious workup was unremarkable. She was diagnosed with uncomplicated malaria and started on antimalarial therapy with a three-day course of artemether 80 mg twice daily and lumefantrine 480 mg twice daily.

**Figure 1:**
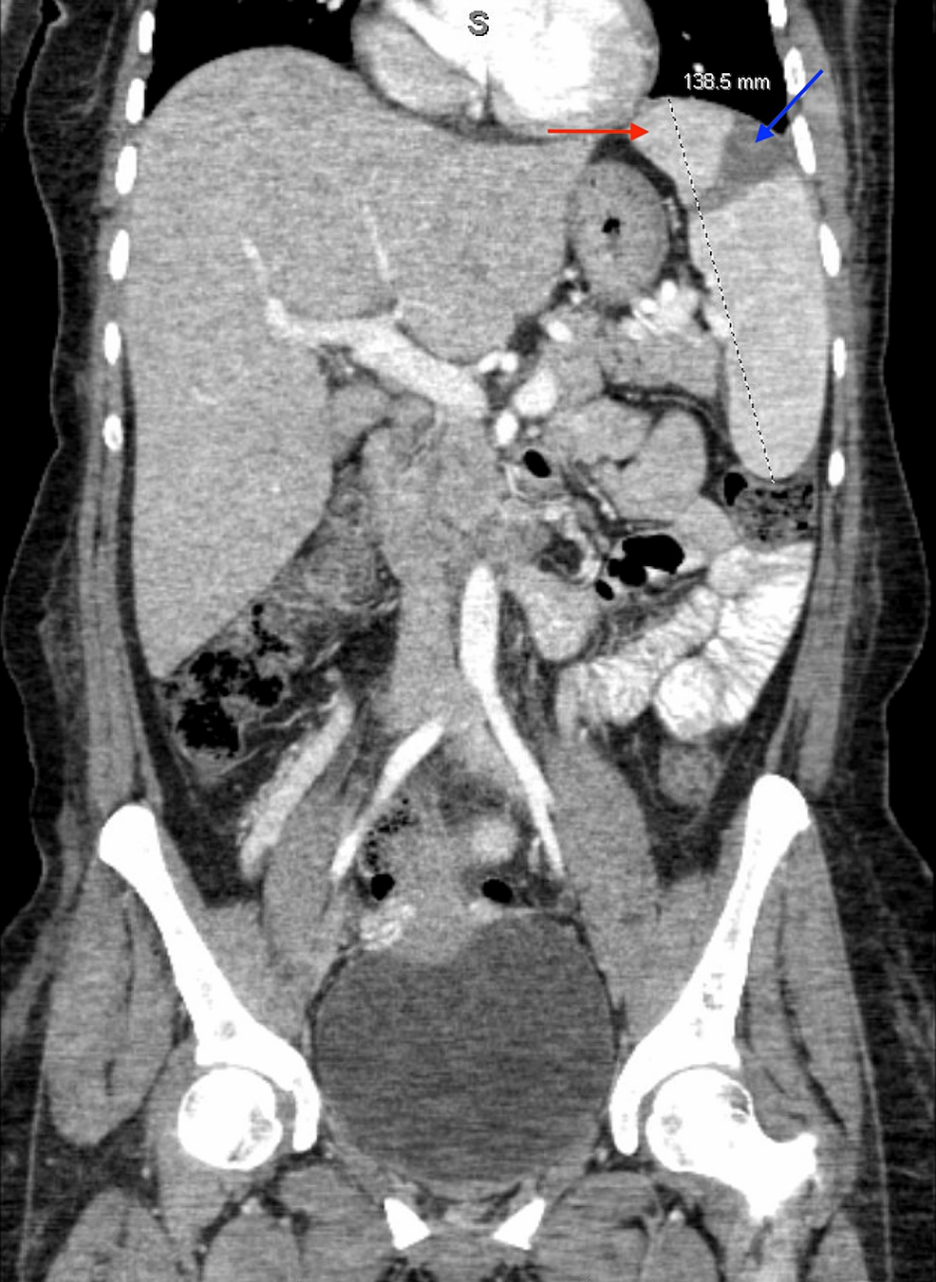
CT scan of the abdomen and pelvis CT scan of the abdomen and pelvis in coronal view demonstrates an enlarged spleen measuring 13.8 cm (red arrow) with a band of hypoattenuation that likely represents an infarction (blue arrow).

The patient had a transient clinical recovery, after which she developed significant fatigue and endorsed left upper quadrant abdominal discomfort and swelling. Abdominal computed tomography (CT) scan demonstrated an enlarged spleen measuring 13.8 cm with a possible splenic infarct (Figure 1). This warranted further investigations, and there was a reasonable concern for HLH.

The patient met the diagnostic criteria for HLH with the following features: fever of 38.6° C, splenomegaly, anemia, thrombocytopenia, hyperferritinemia, hypertriglyceridemia, and a markedly high soluble interleukin-2 receptor CD25 (sCD25) level (Table 1). Disseminated intravascular coagulation was ruled out as a cause of her clinical findings given the normal coagulation profile (prothrombin time, activated partial thromboplastin time, international normalized ratio) and the absence of schistocytes on peripheral smear.

**Table 1:** Laboratory investigations sCD25 - soluble interleukin-2 receptor CD25

	Patient's results before treatment	Patient's results after treatment	Reference range
Hemoglobin (g/dL)	8.1	10.2	12.0 - 15.0
Platelet count (K/uL)	115	324	150 - 450
Absolute neutrophil count (K/uL)	1.20	2.01	1.80 - 7.70
Triglyceride (mg/dL)	612	387	40 - 200
Fibrinogen (mg/dL)	191	206	200 - 450
Ferritin (ng/mL)	1458	639	11 - 307
sCD25 level (pg/mL)	3300.9	N/A	175.3 - 858.2

After completion of her course of antimalarial therapy, our patient had marked clinical improvement, recovery of cell counts, and decline in inflammatory markers. Repeat malaria smear demonstrated a parasite burden of less than 0.1%.

## Discussion

HLH is an inflammatory syndrome that occurs in the setting of pathologic immune system activation and manifests with a characteristic constellation of clinical features. It involves an interplay of genetic, environmental, and clinical factors and has a variable spectrum of disease and presentation.

The pathogenesis of HLH remains poorly understood. Abnormal activation of the immune system is the cornerstone of this syndrome as it allows for excessive inflammation and destruction of tissue. Normally, natural killer (NK) cells and cytotoxic lymphocytes (CTLs) regulate the inflammatory response of macrophages through a negative feedback loop. In HLH, dysfunction of this regulatory pathway creates an environment in which macrophages are not eliminated; they persist in a state of hyperactivation [7-8]. Inappropriately activated macrophages phagocytize host blood cells, including white blood cells, red blood cells, and platelets, in a phenomenon termed hemophagocytosis. Moreover, they infiltrate tissues causing local inflammation and tissue destruction [8-9]. Macrophages, NK cells, and CTLs create a cytokine storm with excessive release of cytokines, including interferon-gamma, tumor necrosis factor-alpha, and interleukins; these factors further propagate inflammation and are responsible for the complications of the syndrome and multiorgan failure [10].

HLH is strongly associated with mutations in genes involved in the immune system. It can occur as an isolated genetic defect or as a component of primary immunodeficiency syndromes [11]. In patients without a known genetic predisposition to HLH, the most identified trigger is an infection. This syndrome has been described in the setting of viral, bacterial, fungal, and parasitic infections. The Epstein-Barr virus is the most implicated pathogen. HLH occurring in the setting of malarial infection is very rare, and literature on this association is limited.

The HLH-2004 diagnostic criteria have been established as the diagnostic standard for the syndrome. It was initially utilized in prospective treatment studies for children with HLH and was later extrapolated to adults and adopted for use in regular clinical practice [1,12]. The diagnosis of HLH can be made if either criterion 1 or 2 is fulfilled (Table 2). Recently, a scoring system termed the "HScore" was validated to estimate the probability of HLH in a patient [13]. In clinical practice, patients in whom there is a high clinical suspicion for HLH are often treated according to modified criteria that are less specific for the syndrome; this is because of the high morbidity and mortality associated with the delay of waiting for the results of genetic or immunologic tests [14-16].

**Table 2:** HLH-2004 diagnostic criteria [12] The diagnosis of HLH can be established if criterion A or B is fulfilled. HLH - hemophagocytic lymphohistiocytosis; NK - natural killer

A. Molecular diagnosis consistent with HLH
B. Diagnostic criteria for HLH fulfilled (five of the eight criteria below)
Fever
Splenomegaly
Cytopenias affecting two or three lineages in the peripheral blood: hemoglobin <90 g/L, platelets <100 x 109/L, neutrophils <1.0 x 109/L
Hypertriglyceridemia (fasting triglycerides ≥ 265 mg/dL) and/or hypofibrinogenemia (fibrinogen ≤1.5 g/L)
Hemophagocytosis in bone marrow, spleen, or lymph nodes
Low or no NK cell activity
Hyperferritinemia (ferritin ≥500 µg/L)
sCD25 (ie, soluble IL-2 receptor) ≥2400 U/mL

Significant overlap exists between features of malaria and HLH, which raises a diagnostic dilemma. Fever, splenomegaly, anemia, thrombocytopenia, and elevated inflammatory markers are often present in patients with malaria and can be explained by parasite-induced cytotoxicity, microvascular disease, and organ sequestration. However, high ferritin, high triglyceride, and low fibrinogen levels are uncommon in malaria and may be suggestive of superimposed HLH. The diagnosis can also be corroborated with immunological testing for sCD25, C-X-C motif chemokine ligand 9 (CXCL9), or NK cytotoxicity assay, which are specific for HLH.

A review of the literature yielded 28 cases of HLH associated with malaria worldwide [17-41]. Most of these cases were reported outside of the United States, given the epidemiology of malaria and its higher prevalence in Sub-Saharan Africa and Southeast Asia [42]. All the reported cases confirmed the diagnosis of malaria by visualization of the parasite on peripheral blood smear or bone marrow biopsy. The diagnosis of HLH was made based on the HLH-2004 criteria. The temporal relationship between the malarial infection and the development of HLH in these cases was sufficient to establish it as the likely trigger. Plasmodium falciparum and vivax are the only two malaria species associated with HLH thus far, with Plasmodium falciparum implicated in most cases. The pathogenesis of HLH in the setting of malaria is unknown, although it likely involves an interplay between a patient's genetic susceptibility and a persistent and inappropriate immune response induced by the parasite.

A portion of patients with HLH improve with early identification and prompt treatment of the trigger. HLH-specific therapy, therefore, is reserved for patients with clinical deterioration or multiorgan failure. HLH-specific therapy typically consists of a combination of etoposide, glucocorticoids, methotrexate, and intravenous immunoglobulin. Among the 28 published cases of HLH associated with malaria, 18 patients (65%) had clinical improvement and resolution of abnormal laboratory findings after typical treatment courses with antimalarials alone [17-33]. Patients who were unresponsive to initial therapy or had clinical decompensation were also treated with immunosuppression. Glucocorticoids were used to treat four patients (14%) [34-37]. Intravenous immunoglobulin was used to treat six patients (21%) [38-41]. Most patients with malaria-associated HLH recovered after antimalarial therapy alone, as did our patient who did not require HLH-specific therapy. All reported patients with HLH-associated malaria recovered, and there were no mortalities.

## Conclusions

HLH is a rare but life-threatening complication of severe infections. Its identification in the setting of malaria is uncommon, although this could partly be due to under-recognition and diagnostic difficulties. Our patient's clinical deterioration after an initial response to antimalarial therapy helped identify this syndrome; this prompted closer monitoring and supportive care, after which she had complete clinical recovery. This case of HLH associated with Plasmodium falciparum malaria serves to add to the existing literature on this association which will allow for a better understanding of this syndrome. Further investigation into this association is needed to help predict at-risk patients and to identify the most effective treatment regimen. Maintaining a high index of suspicion for HLH-associated malaria allows for early identification, close monitoring, and treatment of the underlying cause to improve patient outcomes.
